# *In vitro* degradation of pure Mg in response to glucose

**DOI:** 10.1038/srep13026

**Published:** 2015-08-12

**Authors:** Rong-Chang Zeng, Xiao-Ting Li, Shuo-Qi Li, Fen Zhang, En-Hou Han

**Affiliations:** 1 College of Materials Science and Engineering, Shandong University of Science and Technology, Qingdao 266590, China; 2 State Key Laboratory of Mining Disaster Prevention and Control Co-founded by Shandong Province and the Ministry of Science and Technology, Shandong University of Science and Technology, Qingdao 266590, China; 3 National Engineering Center for Corrosion Control, Institute of Metals Research, Chinese Academy of Sciences, Shenyang 110016, China

## Abstract

Magnesium and its alloys are promising biodegradable biomaterials but are still challenging to be used in person with high levels of blood glucose or diabetes. To date, the influence of glucose on magnesium degradation has not yet been elucidated, this issue requires more attention. Herein, we present pure Mg exhibiting different corrosion responses to saline and Hank’s solutions with different glucose contents, and the degradation mechanism of pure Mg in the saline solution with glucose in comparison with mannitol as a control. On one hand, the corrosion rate of pure Mg increases with the glucose concentration in saline solutions. Glucose rapidly transforms into gluconic acid, which attacks the oxides of the metal and decreases the pH of the solution; it also promotes the absorption of chloride ions on the Mg surface and consequently accelerates corrosion. On the other hand, better corrosion resistance is obtained with increasing glucose content in Hank’s solution due to the fact that glucose coordinates Ca^2+^ ions in Hank’s solution and thus improves the formation of Ca-P compounds on the pure Mg surface. This finding will open up new avenues for research on the biodegradation of bio-Mg materials in general, which could yield many new and interesting results.

Ageing of the population has led to major public health concerns regarding the levels of blood glucose and the prevalence of diabetes, a glucose regulation disorder^1^. Globally, the number of patients with diabetes is predicted to double to 366 million by 2030 from 171 million in the year 2000^2^. In China, the age-standardized prevalence for total diabetes and pre-diabetes were reported to be as high as 9.7% and 15.5%, respectively^3^. Thus, the ageing of the population globally increases the need for biomaterials, which have to face huge challenge to be implanted in the patients with diabetes and pre-diabetes.

There are four major types of materials that are used as biomaterials: metals, ceramics, polymers and their composites^4^. Metallic materials such as stainless steels, titanium alloys, and cobalt-based alloys are state-of-the-art materials in medical implants given their high strength, high ductility, and good corrosion resistance^5^,^6^,^7^,^8^,^9^. However, the risk of adverse effects or local inflammation may increase with long-term implants due to the response of human tissues to foreign metallic implants. In the case of an adverse reaction, a second surgery to remove the implants may be necessary. Conversely, the higher elastic modulus^8^ of implanted biomaterials compared to human bone results in a stress shielding effect.

Magnesium and its alloys have a considerable potential for biological applications given their unique biocompatibility, biodegradability and mechanical properties that are similar to those of human bones^9^,^10^,^11^,^12^,^13^,^14^,^15^. Therefore, the most attractive clinical applications are for cardiovascular intervention and orthopaedic surgeons, despite the fact that some of the early orthopaedic magnesium alloys have not been successful and have fallen into disuse^12^,^16^,^17^,^18^. Magnesium-based materials were introduced as orthopaedic biomaterials in the first half of the 20^th^ century^8^. In recent decades, biomedical magnesium has sparked interested in the scientific community because of the need for advanced biomaterials to support the ageing population and to improve the quality of life. However, excessively fast or uncontrolled degradation of Mg and its alloys is a serious challenge.

The biodegradation of magnesium is fundamentally linked to studies of its corrosion, which depends on the dynamics at the interface between the material and its environment^19^. Biomaterial research has made major strides in this area, and a myriad of studies on the degradation behaviour of Mg and its alloys in physiological solutions are underway^20^,^21^. Blood plasma and intercellular fluid are neutral solutions containing inorganic ions such as Mg^2+^, Ca^2+^, Cl^−^, 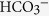 and 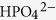 ions^22^,^23^,^24^,^25^,^26^, as well as organic compounds such as amino acids, proteins and glucose^27^. Our previous investigation^22^ and other studies^23^,^24^,^25^ have demonstrated that chloride ions can induce pitting corrosion, while phosphates^25^,^26^ can slow the corrosion rate effectively. Carbonate^19^,^28^ can promote the dissolution of magnesium but can also induce rapid surface passivation due to the precipitation of magnesium carbonate. Sulfate ions^29^,^30^ are more aggressive than chloride in the case of general corrosion. Instead of the expected reduction of corrosion and the slight passivation behaviour in the presence of albumin^27^,^31^,^32^,^33^, an acceleration of the corrosion was observed, depending on the concentration of albumin^34^,^35^. Yang *et al.*^32^ reported that the use of proteins and/or buffered solutions under atmospheric conditions and the use of cell culture conditions all led to different results, and proteins delayed corrosion and altered the ion composition of the solutions. Moreover, glucose has an important influence on the corrosion of metallic alloys, including mild steel, cast iron and Ti alloys in particular. For example, glucose acts as an inhibitor against the corrosion of mild steel^36^ and cast iron^37^ in acidic solutions via both physical and chemical interactions. In addition, glucose can be rapidly absorbed on titanium surfaces, and the absorbed glucose permits ionic diffusion of oxygen to the electrode^38^.

Unfortunately, few studies have focused on the significant influence of glucose on the biodegradation of magnesium. Willumeit^39^ suggested that glucose has least impact on magnesium degradation under atmosphere and cell culture conditions in the presence of O_2_ and CO_2_ by artificial neural networks. Glucose does not necessarily interact with any of the available partners or components (i.e., protein, O_2_, CO_2_, NaCl, NaHCO_3_, CaCl_2_ and MgSO_4_) in the medium. However, the synergistic influence of CO_2_ and glucose on the degradation of magnesium might be ignored, both of which could lower solution pH.

When implanted into the human body, the metal will most certainly be exposed to bodily fluids that contain glucose. It is well known that a higher glucose level may lead to diabetes, and the risks from magnesium biodegradation for patients with diabetes are disconcerting given the growing incidence of this disease^40^. Despite successful implant treatments in healthy persons, implant failures are more likely to occur in those with medically compromising systemic conditions, such as diabetes^41^. Thus, problems inevitably arise in designing materials for biomedical needs in clinical applications when the magnesium would be implanted into humans with higher glucose contents, especially those with diabetes. However, it must be noted that magnesium is predominantly an intracellular ion that plays a key role in the regulation of glucose and insulin metabolism^42^,^43^,^44^. Therefore, investigations into the interaction between biodegradable magnesium and physiological environments with glucose ought not to be neglected. Herein, the *in vitro* corrosion mechanism was compared for pure Mg in both isotonic saline solution (0.9 wt.% NaCl) and Hank’s solution with different glucose contents. The formation of corrosion product varied with the solutions with different glucose contents, and thus the corrosion mechanism needs to be clarified. The results provide a novel knowledge that the unique corrosion behaviour of magnesium should be taken into consideration when magnesium is implanted into a patient with a high glucose level or with diabetes.

## Results

### Influence of glucose on corrosion in saline solution

**Figure 1:**
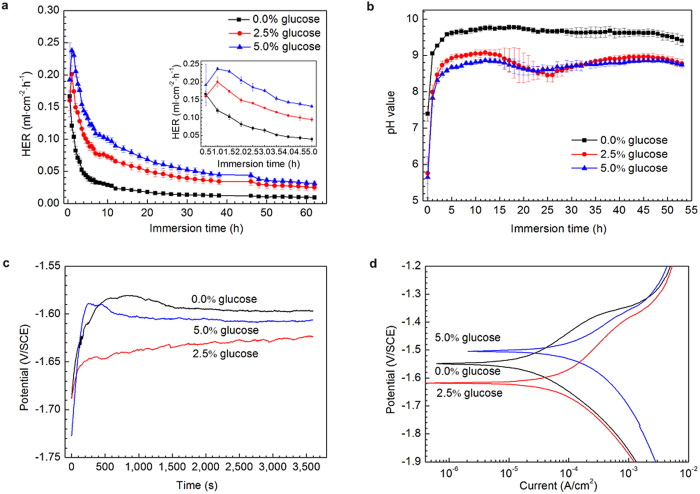
Corrosion and electrochemical measurements of pure Mg in 0.9% NaCl solutions with different glucose contents. (**a**) hydrogen evolution rate. (**b**) solution pH value. (**c**) Open-circuit potential. (**d**) Polarization curves.

Figure 1a depicts the hydrogen evolution rate (HER) of pure Mg exposed to a saline solution with different glucose contents at 37 ± 0.5 °C for 62 h. It was noted that an increase in the glucose content generated a significant change in the HERs. The samples showed a high HER at the start of immersion during the experiment in the absence of glucose and a distinct drop with further immersion. Conversely, HERs in the presence of glucose increased quickly in the initial 1 h of the immersion and then decreased sharply as shown in the inset of Fig. 1a. Most importantly, pure Mg samples exhibited a higher corrosion rate upon addition of glucose. The variation in the solution pH values during the immersion tests in each solution are shown in Fig. 1b. The dissolution of Mg can dramatically enhance the pH value for all of the samples. From approximately 5 h onwards, the pH values did not change much and eventually stabilized. However, adding glucose reduced the pH values. It was also noted that after 15 h of immersion, the pH of the solutions in the presence of glucose slightly dropped, but then it steadily increased from approximately 25 h. This implies that glucose causes the acidity of the solution as the pH value decreases, and then the pH values rise gradually with the formation of a new Mg(OH)_2_ film. Thus, the dissolution and formation of films formed on samples immersed in solutions containing glucose create a dynamic balance until the film is compact.

This result was also supported by the measurement of the open-circuit potential (OCP) of the immersed samples. As shown in Fig. 1c, an increase in the OCP indicates that a corrosion layer formed on the metal. It is worth noting that the different glucose contents resulted in a difference in the *E*_*corr*_. In the absence of glucose, from approximately 500 s, the *E*_*corr*_ stabilized and only slightly fluctuated at approximately 1,000 s. The addition of glucose to the saline solution shifted the *E*_*corr*_ to lower values. The *E*_*corr*_ of the samples tested in 2.5% glucose solutions fluctuated frequently throughout the test, which implies that it was difficult for the sample to reach equilibrium at the electrode/solution interface. In the presence of 5.0% glucose, an abrupt drop in *E*_corr_ at approximately 450 s indicated the occurrence of pitting corrosion on the sample surface. It has been reported that Mg(OH)_2_ corrosion product films formed on pure magnesium surfaces after immersion in NaCl solutions grow rapidly but quasi-passively^45^. Chloride ions promote the dissolution of magnesium and make hydroxide films more active or increase the film-free area. Thus, the fluctuation of the OCP values in the NaCl solution can be attributed to the rupture of corrosion products on the film. However, with the addition of glucose, the potential shifted towards less negative values quickly and fluctuated frequently throughout the test, indicating that the glucose destabilized the formed layer and stopped its growth. Additionally, with the longer immersion times, the corrosion mechanism changed from uniform corrosion to pitting corrosion because of the galvanic effect from the decline of the corrosion potential, as displayed on the curves^46^. When the glucose concentration was 5.0%, the initiation of pitting corrosion for pure Mg started almost as soon as the sample was soaked in the solution. In addition, the OCP results were confirmed by the potentiodynamic polarization curves (Fig. 1d). The current densities for the pure Mg samples in the three solutions (0.0 wt.%, 2.5 wt.%, and 5.0 wt.% glucose) were 2.99 × 10^−5^, 9.37 × 10^−5^, 9.17 × 10^−3 ^A/cm^2^, respectively. The presence of glucose enhanced the current density. The higher the glucose contents were in the saline solution, the faster the corrosion rates were for pure Mg. The results suggest that the addition of glucose increase the corrosion rate of pure Mg in saline solution.

**Figure 2:**
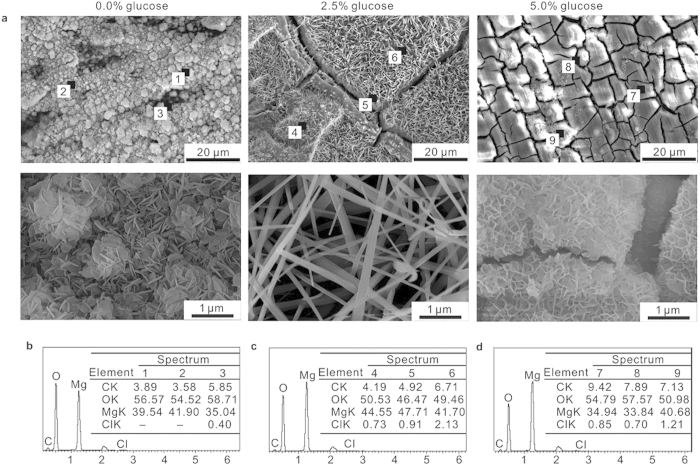
SEM morphologies and corresponding EDS of pure Mg after immersion in 0.9% NaCl solutions with different contents of glucose for 24 h. (**a**) SEM morphologies of pure Mg. (**b**–**d**) EDS of pure Mg in 0.9% NaCl solutions without glucose (**b**), with 2.5% glucose (**c**) and with 5.0% glucose (**d**). The EDS was organized by wt.%.

Figure 2 shows the SEM morphologies and corresponding EDS analysis of the pure Mg samples after immersion in 0.9% NaCl solutions with different contents of glucose for 24 h. The pure Mg samples were subjected to pitting corrosion and corrosion cracks. As shown in Fig. 2a, in the absence of glucose, thick and irregular spherical nano-particles composed of dense, multilateral, flack-like nano-microstructures formed on the sample surface. With the addition of glucose, the appearance of the surface completely altered. With the addition of 2.5% glucose, corrosion products with wide and deep cracks, fractures and corrosion pits covered the sample surfaces, and numerous nano-wires deposited on the entire sample surface. When the glucose concentration was increased to 5.0%, corrosion products with honeycomb-like morphologies were observed on the surface.

Figure 2b–d shows the EDS analysis of the sample after immersion in 0.9% NaCl solutions with different contents of glucose for 24 h. The primary constituents formed on the pure Mg surface after immersion in the different test solutions were C, O and Mg, and trace amounts of Cl. The presence of C mainly came from the carbon sputtering process. The result indicates that the corrosion products are composed of magnesium hydroxide. Moreover, the existence of higher Cl^−^ contents on the surface (spectrum 4–9) in the presence of glucose than in the absence of glucose (spectrum 3) indicates that the addition of glucose promotes absorption and accumulation on the surface, resulting in more severe corrosion.

**Figure 3:**
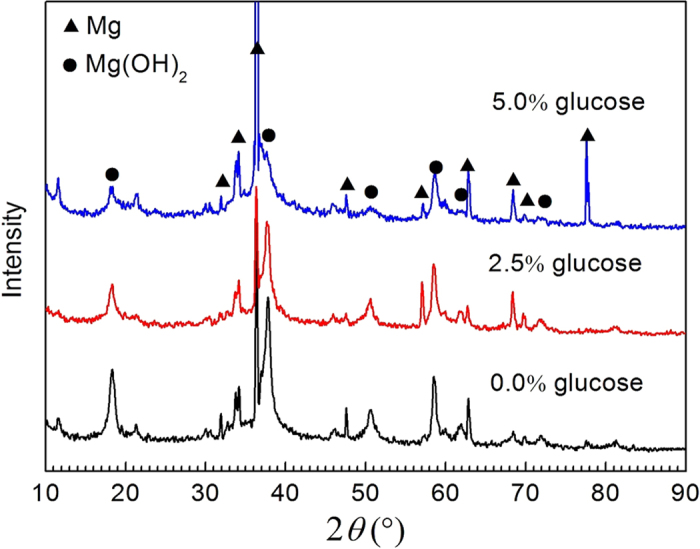
XRD patterns of pure Mg after immersion in 0.9% NaCl with different glucose contents for 72 h.

Figure 3 shows the XRD patterns of the samples after exposure to the solution for 3 days. In addition to the dominant peaks corresponding to the Mg phase, some peaks at approximately 2*θ* = 18, 38, 51, 58, 62, 72° correspond to the diffraction peaks of Mg(OH)_2_ that can be clearly detected in all of the patterns. However, with an increase in the glucose concentration, the peak intensity of Mg(OH)_2_ decreased compared to situations with no glucose. This indicates that fewer Mg(OH)_2_ corrosion products deposited on the surface of the pure Mg during the corrosion process. The XRD patterns demonstrate that glucose seems to inhibit the formation of the Mg(OH)_2_ film, which promotes the corrosion of pure Mg.

To determine the corrosion mechanism for pure magnesium in the presence of glucose, further investigations were performed. Glucose possesses both alcohol and aldehyde groups. In view of the similar structures between glucose and mannitol, mannitol was used as a control to determine the glucose group responsible for the corrosion behaviour of pure Mg in the 0.9% NaCl solution.

**Figure 4:**
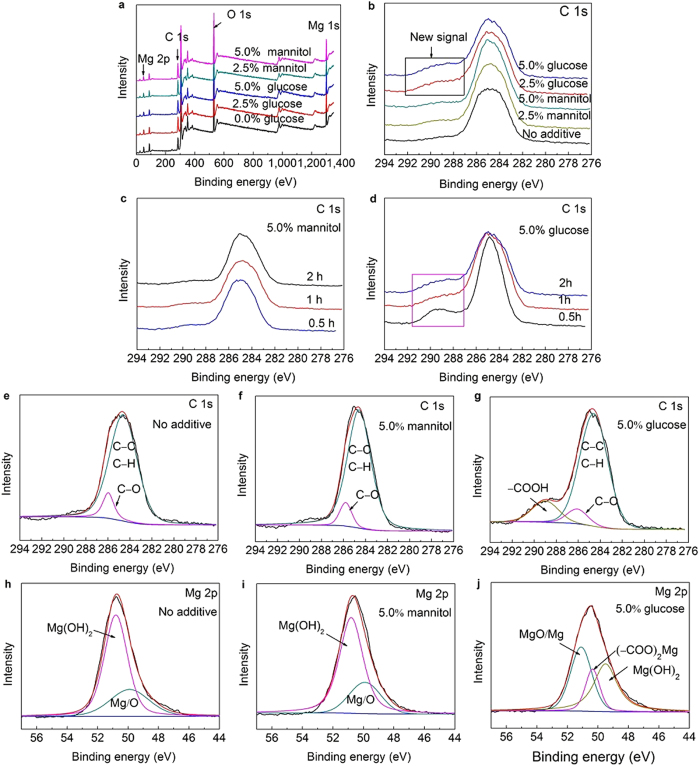
XPS analysis of a pure Mg surface after immersion in 0.9% NaCl solutions without and with glucose or mannitol for various periods. (**a**,**b**) The entire range of the binding energy survey (**a**) and C 1s spectra (**b**) for sample surfaces after immersion in 0.9% NaCl solutions without and with glucose or mannitol for 2 h. (**c**,**d**) C 1s spectra of samples immersed in 0.9% NaCl solutions with mannitol (**c**) and with glucose (**d**) for different amounts of time. (**e**–**j**) curve fitting of C 1s spectra (**e**–**g**) and Mg 2p spectra (**h–j**) for samples after immersion in 0.9% NaCl solutions with no additive (**e**,**h**) and with 5.0% mannitol (**f**,**i**) and 5.0% glucose (**g**,**j**) for 2 h.

The XPS analyses of the pure Mg surface after immersion in a 0.9% NaCl solution without and with glucose or mannitol are displayed in Fig. 4a–j. Figure 4a shows the whole range of the binding energy survey on all sample surfaces. The results reveal that there was no significant difference in the chemical compositions of the corrosion products, which mainly consist of C, O, and Mg elements, among the three solutions. To obtain more information on the nature of the surface, high-resolution XPS data for C 1s and Mg 2p were also collected.

The C 1s spectrum (Fig. 4b) clearly depicts the samples immersed in 0.9% NaCl solution without and with different additives (glucose or mannitol) for 2 h. A new peak near 289 eV can be observed upon addition of glucose, while no new signal was observed in the absence of glucose and mannitol. This indicates that the glucose transformed into another substance, and a new product formed on the sample surface after adding glucose. However, the addition of mannitol did not change the corrosion products formed on sample surface.

To further demonstrate these results, XPS analyses were performed for the samples immersed in 0.9% NaCl with glucose and mannitol for different times. Figure 4c,d show the C 1s spectra of the samples immersed in 0.9% NaCl solutions with mannitol and glucose, respectively, for different amounts of time. The results are in good agreement with the results of the previous test. It is interesting to note that a new signal appeared after immersion for 0.5 h (Fig. 4d), implying that the glucose transformation into another substance occurred in a short time. In contrast, the C 1s spectra did not seem to be affected by adding mannitol (Fig. 4c).

Figure 4e–g designate the curve fits of the C 1s spectra for the samples after immersion in 0.9% NaCl solutions without and with different additives (glucose or mannitol) for 2 h. In the solution with no additive (Fig. 4e) and in the presence of mannitol (Fig. 4f), the C 1s spectra can be split into two peaks, indicating three chemical forms of the C atoms on the sample surface. The main peak can be attributed to a hydrocarbon species (C−H/C−C) with a characteristic binding energy of 284.6 eV^47^. The other component, at 285.9 ± 0.1 eV, may be associated with the presence of C−O. However, the high resolution of the C 1s spectrum displays a noticeable difference when the samples were immersed in the presence of 5.0% glucose (Fig. 4g). In addition to the C−H/C−C and C−O groups, a new peak at 289.0 eV was observed that can be associated with the O−C=O−group^48^. It can be seen clearly in Fig. 4d that when the samples were immersed in 0.9% NaCl with 5.0% glucose for 0.5 h, the signals for the C−H/C−C and C−O groups were very strong. This indicates that large proportion of this group came from glucose and that glucose adsorption on the sample surface was rapid. The intensity of this group decreased with prolonged immersion, which provides evidence that the glucose continued to transform with extended immersion times.

Figure 4h–j demonstrates the curve fits of the Mg 2p spectra for the samples after immersion in 0.9% NaCl solutions without and with different additives (glucose or mannitol) for 2 h. With no additive and in the presence of mannitol, the Mg 2p spectra can be split into two peaks; one peak can be attributed to Mg(OH)_2_, and the other can be attributed to Mg/O. However, three peaks were obtained in the presence of 5.0% glucose, which can be attributed to MgO/Mg, (−COO)_2_Mg, and Mg(OH)_2_. The presence of (−COO)_2_Mg implies that the −COOH formed during the transformation of glucose, leading to the attack of the Mg(OH)_2_ precipitate and improvement in the formation of (−COO)_2_Mg.

The XPS analysis shows that the formation of −COOH from glucose is the dominant factor for the increased corrosion. In addition, the functional group of glucose in the study is from Carbon #1. An aqueous sugar solution contains only 0.02% glucose in the chain form; the majority of the structure is in the cyclic chair form (D-glucose). Carbon #1 is the anomeric carbon and the centre of a hemiacetal functional group that possesses both oxygen and an alcohol group. The aldehyde group in glucose is very active and can be transformed into a carboxyl group (−COOH). Then, the carboxyl group can attack the film of corrosion products on a pure Mg surface and accelerate the corrosion process.

### Influence of glucose on corrosion in Hank’s solutions

**Figure 5:**
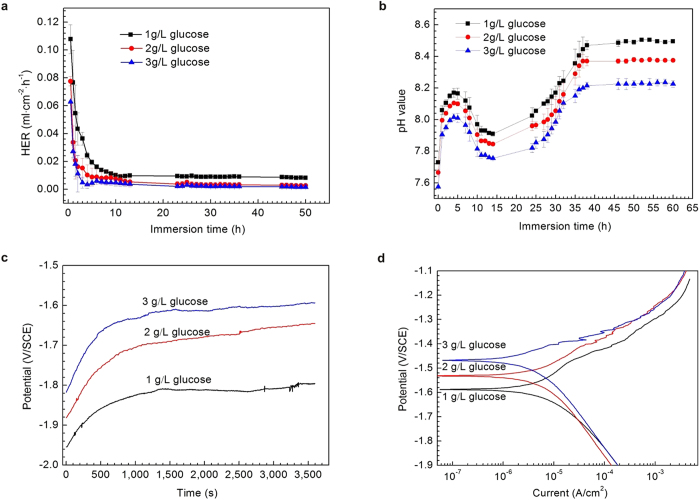
Corrosion and electrochemical measurements of pure Mg in Hank’s solutions with different glucose contents. (**a**) hydrogen evolution rate. (**b**) solution pH value. (**c**) Open-circuit potential. (**d**) Polarization curves.

Figure 5a shows hydrogen evolution rate (HER) of pure Mg exposed to Hank’s solutions with different glucose contents (1 g/L, 2 g/L, 3 g/L) at 37 ± 0.5 °C for 50 h. The HERs in the first 1 h were very high and dripped significantly during the following several hours, and then stabilized with extending immersion time. In contrast to the results in the 0.9% NaCl solutions discussed above, increasing glucose content in Hank’s solutions decreased the corrosion rate of pure Mg samples. After 50 h of immersion, the HERs were 0.0082 ± 0.00018 ml • cm^−2^ • h^−1^ for 1 g/L glucose, 0.0026 ± 0.0011 ml • cm^−2^ • h^−1^ for 2 g/L glucose and 0.0016 ± 0.00024 ml • cm^−2^ • h^−1^ for 3 g/L glucose, respectively. Additionally, note that the corrosion rate (3 g/L) show an increasing trend from 4 h to 6 h, which can be attributed to the existence of small amount of gluconic acid originated from glucose.

The curves of pH values as a function of immersion time in Hank’s solution with different glucose contents (Fig. 5b) are different from that in saline solution (Fig. 1b). A similar fluctuating trend was simultaneously observed in all testing conditions. At the beginning of immersion, the dissolution of pure Mg resulted in a rapid increase in pH for all the samples, and then a markedly decrease in pH, which can be ascribed to the transformation of glucose into gluconic acid discussed above. With the extension of immersion, it started to rise again, and then finally balanced at 8.50 ± 0.007 (1 g/L), 8.38 ± 0.007 (2 g/L) and 8.22 ± 0.021 (3 g/L), respectively.

The open circuit potential (OCP) of the pure Mg samples in Hank’s solutions with different glucose contents (1 g/L, 2 g/L and 3 g/L) is shown in Fig. 5c. A more noble balance potential was observed for the sample in Hank’s solution with increasing glucose content. The increase in OCP (Fig. 5c) over immersion time indicates the growth of a corrosion product layer. The higher OCP indicates a more compact and protective corrosion product layer formed on the samples. Therefore, the higher OCPs for the samples in Hank’s solutions with higher glucose contents designates that the introduction of glucose led to the formation of a more compact corrosion product layer. At the end of the OCP tests, the potentiodynamic polarization curves were immediately measured. The current densities (Fig. 5d) for the pure Mg samples are 3.62 × 10^−6^ A/cm^2^ for 3 g/L glucose, 4.52 × 10^−6^ A/cm^2^ for 2 g/L glucose, slightly lower than 6.82 × 10^−6^ A/cm^2^ for 1 g/L glucose. The results suggest that an increase in glucose content enhanced the corrosion resistance of pure Mg in Hank’s solution, which was in pronounced accordance with HERs.

**Figure 6:**
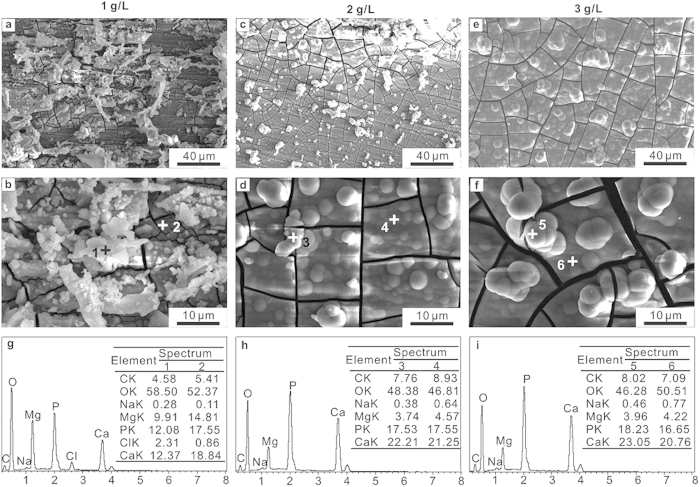
SEM morphologies (**a**–**f**) and corresponding EDS (**g**–**i**) of pure Mg after immersion in Hank’s solutions with different glucose contents for 72 h. (**a**,**b** and **g**) 1 g/L. (**c**,**d** and **h**) 2 g/L. (**e**,**f** and **i**) 3 g/L. The EDS was organized by wt.%.

Figure 6 illustrates the SEM morphologies and corresponding EDS analysis of the pure Mg samples, immersed for 72 h in Hank’s solutions with different contents of glucose (1 g/L, 2 g/L and 3 g/L). The SEM images (Fig. 6a–f) reveal that corrosion products with various morphologies formed on the sample surfaces after immersion in different test solutions. In the solution with 1 g/L glucose (Fig. 6a,b), the corrosion products deposited on the surface inhomogeneously. While in the solution with 2 and 3 g/L glucose, a uniform and compact corrosion layer with some randomly distributed white spherical particles were observed on samples surface (Fig. 6c–f). The presence of C, O, Mg, P, Ca and less amount of Na in the EDS analysis (Fig. 6g–i) indicates the possible existence of Mg(OH)_2_ and Ca-P compounds on the corrosion product layer. A trace of Cl^−^ ions (Fig. 6g) was detected on the sample immersed in Hank’s solution with 1 g/L glucose, which might lead to the dissolution of Mg(OH)_2_ and the formation of incomplete corrosion product layer. A decrease in Mg content and a significant increase in Ca content (Fig. 6h,i) with increasing glucose content in Hank’s solution imply that the glucose promoted the preferential formation of Ca-bearing precipitates, which resulted in the formation of a full and compact corrosion product layer.

**Figure 7:**
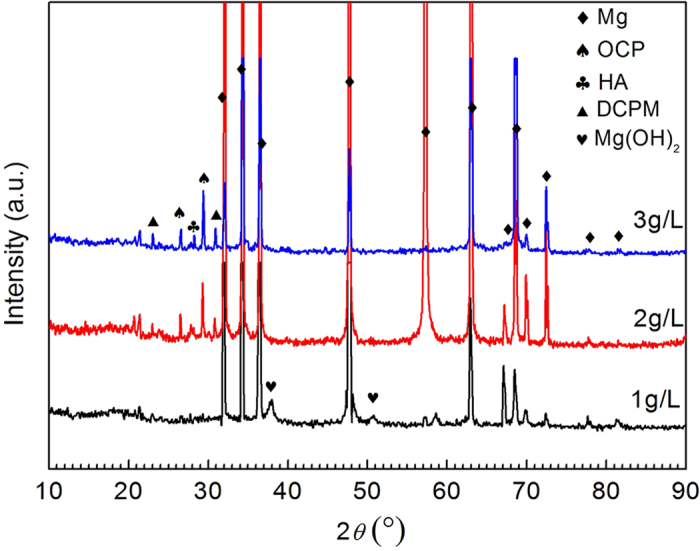
XRD patterns of pure Mg after immersion in Hank’s with different glucose contents for 72 h.

The XRD results (Fig. 7) of pure Mg samples after immersion in Hank’s with different glucose contents reveal the peaks of Mg, Mg(OH)_2_ and Ca-P compounds, i.e., OCP (Ca_8_H_2_(PO_4_)_6_·5H_2_O), DCPM (Ca(H_2_PO_4_)_2_·H_2_O) and a less amount of HA (Ca_10_(PO_4_)_6_(OH)_2_). Obviously, Mg(OH)_2_ were detected at approximately 2*θ* = 38 and 51° only in the Hank’s solution with a glucose content of 1 g/L. It is noting that the intensities of OCP, DCPM and HA increased with the addition of glucose. The XRD results demonstrate that an increase in glucose content in Hank’s solution promoted the formation of calcium phosphate products and an improvement in the corrosion resistance. The results are good consistent with the SEM images and EDS analysis.

According to the above results, glucose promoted the formation of the Ca-P precipitates on the sample surface immersed in Hank’s solution, which can be accounted for the decreasing corrosion rate with increasing glucose content in immersion testing and polarization measurement, and also resulted in a more uniform and compact corrosion product film formed on the sample surface.

## Discussion

**Figure 8:**
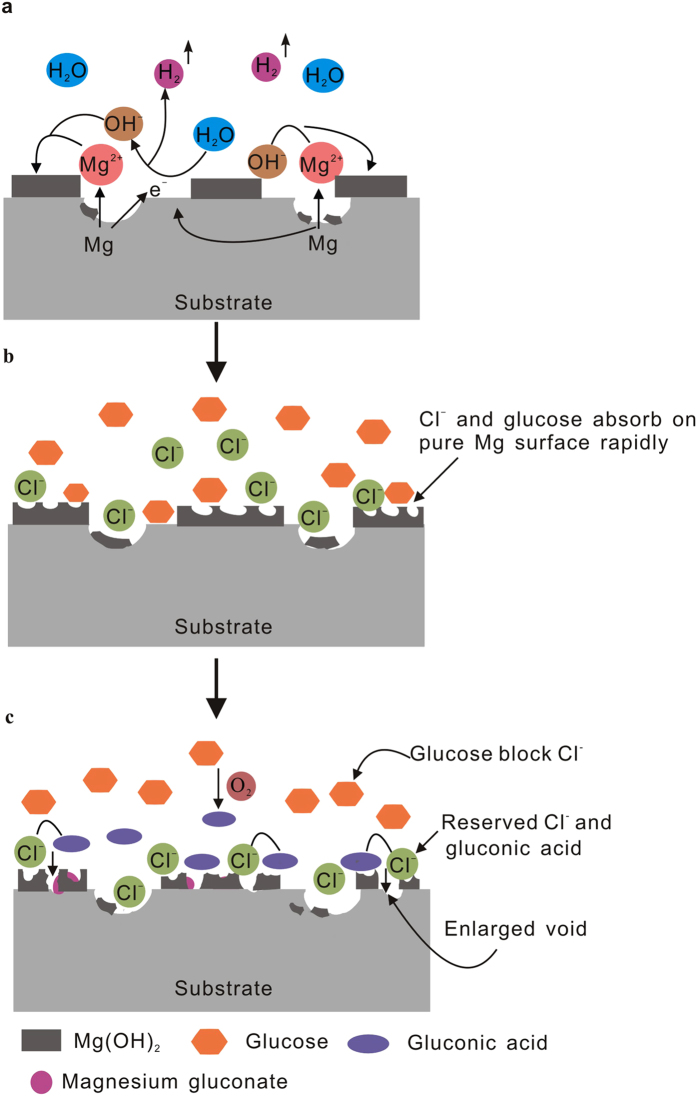
Schematic illustration of corrosion process of pure magnesium during exposure to 0.9% NaCl solution with glucose.

**Figure 9:**
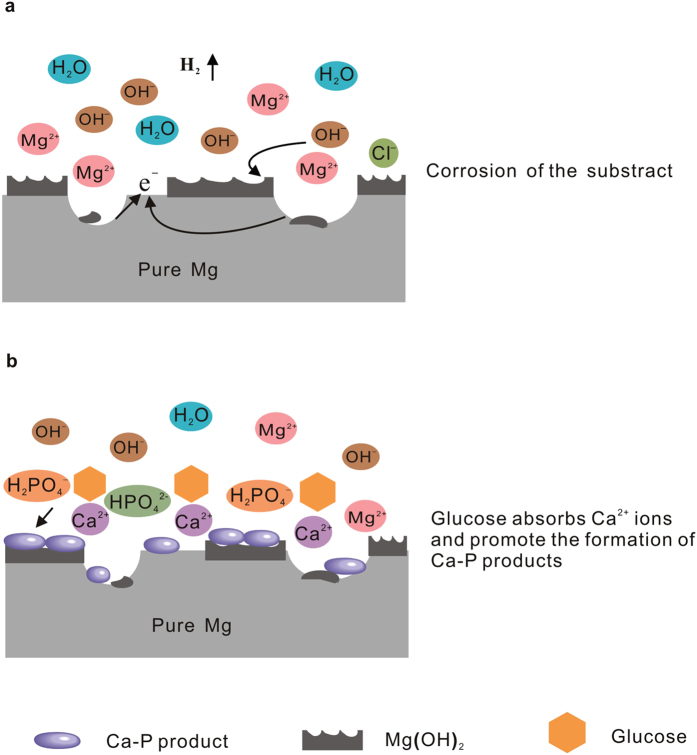
Schematic illustration of corrosion process of pure magnesium during exposure to Hank’s solution with glucose.

In present study, the effects of glucose on the corrosion behaviour of pure Mg in saline and Hank’s solutions were investigated, respectively. However, the results exhibited a significant difference on corrosion behaviours of pure Mg between the two kinds of solutions. Glucose accelerates corrosion of pure Mg in saline solution, while retards the corrosion of pure Mg in Hank’s solution due to the influence of the species such as Ca^2+^ and phosphate ions in Hank’s solution. Based on the above discussions, schematic illustrations of the different corrosion mechanisms for pure Mg in the saline and Hank’s solutions were presented in Figs 8 and 9.

Magnesium is very active in aggressive saline solutions. Once soaking in a solution, the Mg specimens quickly dissolved and released a massive amount of Mg^2+^ ions, alkaline hydroxyl anions and H_2_ gas (Fig. 8a). The chemical reactions follow as


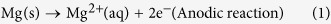



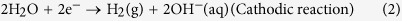



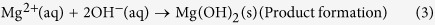


According to reaction (4), a partially protective Mg(OH)_2_ film forms on the Mg sample surface due to the anodic dissolution of magnesium. In the presence of both Cl^−^ ions and glucose, a large amount of Cl^−^ ions and glucose are rapidly absorbed on the pure Mg surface. The Cl^−^ ions are small enough to displace water molecules in the hydrogen sheath and transform the magnesium hydroxide film into the more soluble MgCl_2_ by reaction (4), which promotes the dissolution of magnesium^49^. The dissolution and formation of the corrosion product film will dynamically balance when the film is compact and stops thickening (Fig. 8b).


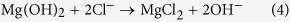


However, the amount of absorbed glucose does not reduce the voids formed on the sample surface or prevent attack from Cl^−^ ions, but it transforms quickly into a new substance, as determined from XPS analysis. First, the aldehyde group of glucose (CH_2_OH(CHOH)_4_CHO) is active and can result in the formation of gluconic acid (CH_2_OH(CHOH)_4_COOH) by oxidizing under certain conditions. Then, the gluconic acid, like the aggressive Cl^−^ ions, can also destroy the Mg(OH)_2_ film, thus accelerating the corrosion process and supporting the precipitation of magnesium gluconate ((CH_2_OH(CHOH)_4_COO)_2_Mg) on magnesium surfaces. Furthermore, glucose can prevent the Cl^−^ ions from breaking away from the sample surface given its strong capacity for adsorption and adhesion. Hence, concerted attacks from the reserved Cl^−^ ions and gluconic acid lead to enlarged voids on the Mg(OH)_2_ film and an open path for the solution to pass through the pores into the substrate, which increases the dissolution activity.

As a result, in the presence of glucose, the samples are subjected to more serious corrosion attacks, and less of the Mg(OH)_2_ film will be preserved on the sample surfaces. This is consistent with the results of XRD analyses. The following additional reactions are caused by the addition of glucose (Fig. 8c)


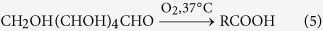



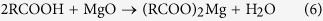



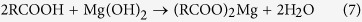


where R is the 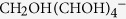 group from CH_2_OH(CHOH)_4_CHO.

On the contrary, the corrosion mechanism of pure magnesium in Hank’s solution with glucose is different from that in saline solution. In the first stage (Fig. 9a), the Mg dissolves and Mg^2+^ ions are released from the substrate. The increase of pH on the Mg surface rapidly results in the formation of Mg(OH)_2_ due to reactions (1)–(3) discussed above. In the second stage, the existence of absorbed Cl^−^ ions and glucose in Hank’s solution can also destroy the formed layer according to reactions (4), (6) and (7). Spontaneously, the adsorption of glucose on the Mg surface is helpful for the accumulation of Ca^2+^ ions on the Mg surface due to the chelation of glucose with Ca^2+^ ions as follows


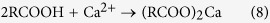


In the third stage, as shown in Fig. 9b, the calcium phosphate begins to nucleate, the absorbed Ca^2+^ ions reacts with 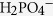 and 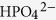 ions in Hank’s solution to form a variety of calcium phosphate precipitates, i.e., OCP, DCPM and HA (Fig. 7). Following reactions may occur


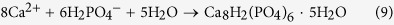



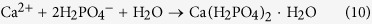



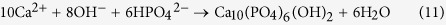


Interestingly, increasing glucose contents in Hank’s solution promote the formation of the Ca-P precipitates, and thus the corrosion resistance of pure Mg. As for the reason, glucose belongs to a kind of polyol aldehyde, which can coordinate with metal ions in aqueous solution depending on its polyhydroxy units. Here, glucose molecular coordinating with Ca^2+^ ions in Hank’s solutions were absorbed on the pure Mg surface. From the above discussions, though glucose transforming into gluconic acid with ionization groups (carboxyl) makes the Mg surface negatively charged. Ca^2+^ ions in Hank’s solutions compete with other ions e.g. Mg^2+^ ions and coordinate with the negatively charged group (carboxyl), reducing the net charges from the molecular of gluconic acid. And consequently, all of the negative charges will be coordinated by the Ca^2+^ ions until the charge neutralization, and hereby the absorption reaches a saturated. As a result, the coordination of Ca^2+^ ions with glucose, absorbed on the pure Mg surface due to high absorbability of glucose, is beneficial for the formation of the Ca-P compounds due to the Ca^2+^ ions reacting with the H_2_PO_4_^−^ and HPO_4_^2−^ ions in Hank’s solution.

It should be pointed out that our findings are different from what is reported by Willumeit *et al.*^39^, who claimed that glucose did not necessarily interact with any available species and had lowest impact on the degradation of magnesium under physiological conditions. This addition of glucose results in a remarkable decrease in both solution pH and corrosion resistance of pure Mg in saline solutions, while an increase in corrosion resistance in Hank’s solutions. In both saline solution and Hank’s solution, the significant decrease in pH of the solution, caused by the introduction of glucose, is ascribed to that glucose is transformed into gluconic acid and promotes absorption of Cl^−^ ions on sample surface after a period of immersion time. As a result, glucose accelerates the corrosion of pure Mg in saline solution, whereas lower the corrosion rate of pure Mg in Hank’s solution due to the coordinating with Ca^2+^ ions and absorbing on the pure Mg surface for glucose in Hank’s solution, and thus promoting the formation of the Ca-P precipitates by the columbic force of the Ca^2+^ and 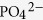 ions.

In summary, the work presented here shows the dramatic results of the addition of glucose into saline solutions and Hank’s solutions. Glucose in both saline and Hank’s solutions performs two functions: lowering the pH of solution due to the transformation into gluconic acid and improving the formation of the Ca-P layer due to the chelating with Ca^2+^ ions. Namely, addition of glucose accelerates the corrosion of pure Mg in saline solution, whereas lowers the corrosion rate of pure Mg in Hank’s solution. This investigation provides a model with which to study the effect of glucose on the corrosion behaviour of magnesium. With a deeper knowledge of these corrosion processes, we can look forward to understanding the corrosion mechanism of magnesium and its alloy as implants in persons with high concentrations of glucose or with diabetes in further clinical research.

## Methods

### Sample preparation

Commercially available pure Mg (99.97% purity) ingots, fabricated by Guangling Magnesium Industry Science and Technology Co. Ltd., were used for the tests. The as-cast metals were cut by electrical discharge machining (EDM) into plates with dimensions of 20 mm × 20 mm × 3 mm. The working surface was sequentially polished with silicon carbide paper from 400 to 2000 grit, cleaned with acetone and ethyl alcohol, and finally dried with warm air. The number of the plates is enough to ensure the measurement performed in triplicate.

### Immersion test

The immersion tests were carried out in various solutions at 37 ± 0.5 °C. Two kinds of solutions, 0.9 wt.% NaCl solutions with glucose (0.0 wt.% (0 g/L), 2.5 wt.% (25 g/L), 5.0 wt.% (50 g/L)) and Hank’s solutions with glucose (1 g/L, 2 g/L, 3 g/L) were used as the test mediums. The saline solution (0.9 wt.% NaCl) was selected with such high glucose contents that the remarkable influence of glucose on corrosion of pure Mg can be clarified in a chemical sense, and also can expel the effect of the other species that may interfere the result of experiments. Based on the study, Hank’s solutions with glucose were used to study the influence of glucose on corrosion behaviour of pure Mg in the simulated body fluid. In the hydrogen evolution test, the ratio of the solution volume to the sample surface was 30 ml/cm^2^, and the temperature was maintained at 37 ± 0.5 °C during the experiments. The samples were soaked into beakers with different solutions. Hydrogen gas was carefully collected from the specimen surface using a funnel placed over the specimens. The hydrogen evolution rate was *v*_*H*_ = *v*/*st* (where *v* is the hydrogen evolution volume (ml), *s* is the sample area exposed to solution (cm^2^), and *t* is the immersion time (h)). The pH values of the solution were recorded at different intervals by a pH meter (PHS-400 type). Each measurement was performed in triplicate.

### Electrochemical testing

Electrochemical measurements were performed on an electrochemical station with a three-electrode system (PARSTAT 2273). A saturated calomel electrode (SCE) and a platinum plate were used as the reference and counter electrodes, respectively. The exposed area of the working electrode to the solution was 1 cm^2^. The experiments were carried out in 0.9 wt.% NaCl solutions with glucose (0.0 wt.% (0 g/L), 2.5 wt.% (25 g/L), 5.0 wt.% (50 g/L)) and Hank’s solutions with glucose (1 g/L, 2 g/L, 3 g/L). All of the electrochemical measurements were performed at ambient temperature. The open circuit potential (OCP) measurements were begun immediately after the specimen was immersed in the solution and lasted for 1 h. After taking the OCP measurements, Tafel curves were scanned at a scan rate of 2 mV·s^−1^. The corrosion rate of the samples was calculated from the curve fit of the Tafel curves.

### Surface analysis

The surface morphologies of the corroded surfaces were discerned using field emission scanning electron microscopy (FE-SEM, NOVA NANOSEM-450). The chemical compositions of the corroded surfaces were probed using an attached energy-dispersive X-ray spectrometer (EDS, JXA-8230). Every sample was sprayed with carbon prior to each test.

The surface functional groups of the samples were detected using Fourier transform infrared spectroscopy (FT-IR, TENSOR-27) in a wavenumber range from 500 to 4000 cm^−1^ at room temperature. The phases of corrosion products were identified using an X-ray diffraction diffractometer (XRD, D/Max 2500PC) with Cu target (*λ* = 0.154 nm).

The chemical compositions of the corrosion-product films on samples immersed in 0.9 wt.% NaCl solutions with glucose (0.0 wt.% (0 g/L), 2.5 wt.% (25 g/L), 5.0 wt.% (50 g/L)) were also probed using X-ray photoelectron spectroscopy (XPS, ESCALAB 250), and the data were taken after 30 s of ion etching. The binding energies were referenced to adventitious carbon (284.60 eV). The data were processed with XPS PEAK 4.1 software and plotted with Origin software.

## Additional Information

**How to cite this article**: Zeng, R.-C. *et al.* *In vitro* degradation of pure Mg in response to glucose. *Sci. Rep.* **5**, 13026; doi: 10.1038/srep13026 (2015).
